# Iron Photoredox Catalysis–Past, Present, and Future

**DOI:** 10.1021/jacs.3c01000

**Published:** 2023-04-20

**Authors:** Lisa H. M. de Groot, Aleksandra Ilic, Jesper Schwarz, Kenneth Wärnmark

**Affiliations:** Centre for Analysis and Synthesis, Lund University, Lund SE-22100, Sweden

## Abstract

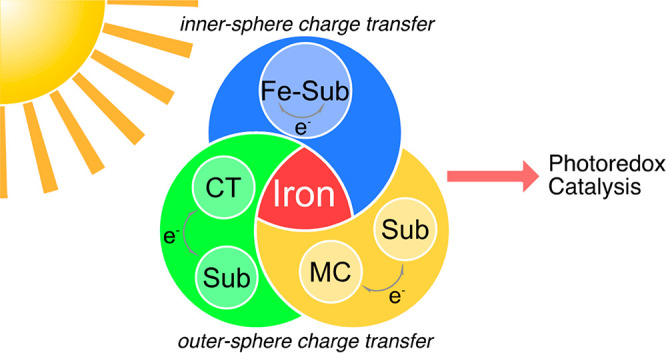

Photoredox catalysis of organic reactions driven by iron has attracted substantial attention throughout recent years, due to potential environmental and economic benefits. In this Perspective, three major strategies were identified that have been employed to date to achieve reactivities comparable to the successful noble metal photoredox catalysis: (1) Direct replacement of a noble metal center by iron in archetypal polypyridyl complexes, resulting in a metal-centered photofunctional state. (2) In situ generation of photoactive complexes by substrate coordination where the reactions are driven via intramolecular electron transfer involving charge-transfer states, for example, through visible-light-induced homolysis. (3) Improving the excited-state lifetimes and redox potentials of the charge-transfer states of iron complexes through new ligand design. We seek to give an overview and evaluation of recent developments in this rapidly growing field and, at the same time, provide an outlook on the future of iron-based photoredox catalysis.

## Introduction

### General Introduction to Photoredox Catalysis

Visible-light-mediated photoredox catalysis is a field that has progressed very rapidly throughout recent years.^1−4^ By using molecules capable of harvesting light, commonly referred to as photoredox catalysts (PCs), that enable a subsequent bimolecular or inner-sphere electron transfer mechanism, highly reactive open-shell intermediates are generated, which can partake in reactions.^5^ Both of these reaction modes result in efficient and useful methods for driving a wide range of organic reactions.^3^ A significant advantage of photoredox catalysis is that photons are capable of providing sufficient amounts of energy to effect the desired reactivities, while omitting high temperature or other harsh conditions.^5^ As a result, this type of chemistry has been used not only to improve many reactions in organic chemistry but also to introduce new reactivity.^6−8^

In general, the employment of visible light is of particular interest, as it constitutes the spectral region where the Sun’s irradiance is highest.^9^ The use of low-energy light for catalysis also leads to fewer undesired side reactions caused by unwanted excitation of organic additives, substrates, and products.^1^ Therefore, it is important to design and use PCs that have strong absorption in the visible region of the spectrum, sufficiently long lifetimes (ns−μs) of their excited states (ESs) to engage in productive, diffusion-controlled bimolecular quenching reactions as well as favorable reversible redox chemistry of their ground states (GSs) and ESs. In addition, a high photostability of the PC is desirable to enable catalysis with a high turnover number (TON).^10^ A variety of both organic and transition metal (TM)-based PCs meet these requirements and have thus been used in a wide range of reactions.^3,11−13^ However, especially TM complexes have been dominating the field to date.^11^ This can be mainly ascribed to the fact that ligand modification and/or choice of counterion enables facile tunability of the absorption wavelength and photoredox properties over a wide range.^14,15^ An additional aspect that is in favor of using TM-PCs is the fact that these often give access to different oxidation states that can be exploited to provide the necessary thermodynamic driving force to complete the catalytic cycle.^14^

**Figure 1:**
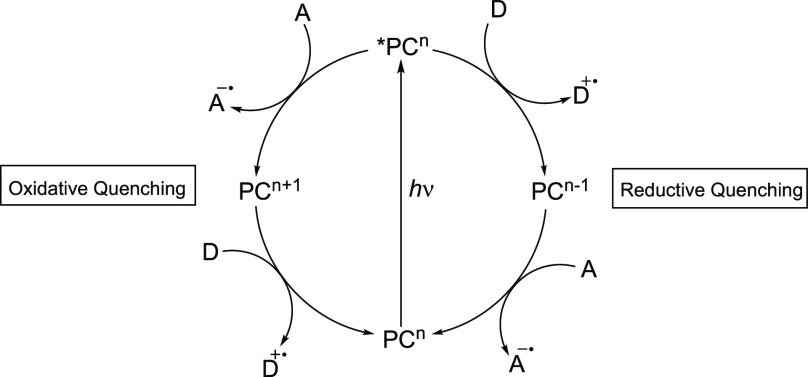
General mechanism of photoredox catalysis via SET; D = (sacrificial) electron donor, A = (sacrificial) electron acceptor, PC = photoredox catalyst.

A general scheme exemplifying how photoredox catalysis frequently operates is illustrated in Figure 1. In the case of conventional intermolecular photoredox catalysis, the PC is excited upon absorption of light. For TM complexes, this often occurs by excitation of an electron from a metal-centered (MC) orbital to a ligand molecular orbital, commonly referred to as a metal-to-ligand charge transfer (MLCT), or vice versa from a ligand molecular orbital to an MC orbital, also known as a ligand-to-metal charge transfer (LMCT). The resulting excited PC (*PC), usually a strong reductant and/or oxidant, can then be reductively quenched by an electron donor or oxidatively quenched by an electron acceptor via single electron transfer (SET).^3^ Electron donors and acceptors can either be sacrificial in nature, giving access to a GS in a different oxidation state, or a substrate/reactant. Following the quenching of the ES, the reduced or oxidized PC is capable of participating in another electron transfer to or from an acceptor or donor molecule, respectively, restoring the original GS.^3^

### The Kinetics of Photoredox Catalysis

**Scheme 1:**
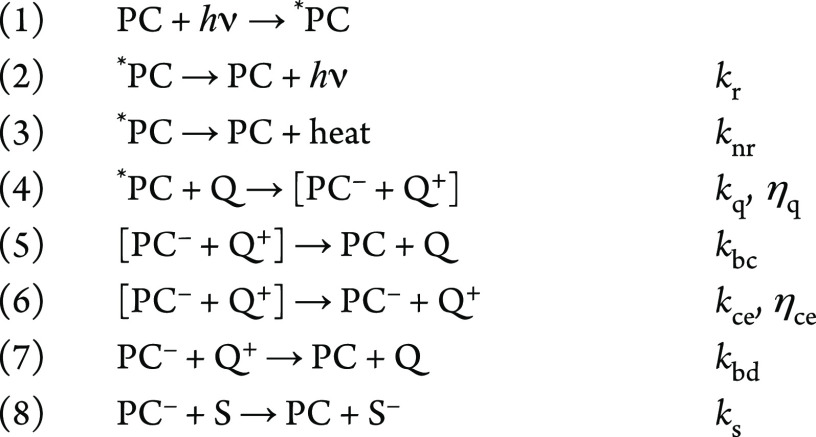
Steps That Commonly Occur upon Light Absorption by a TM-PC and Reductive Quenching^16^ Q = quencher, S = substrate, *k*_r_ = rate of radiative decay, *k*_nr_ = rate of nonradiative decay, *k*_q_ = rate of quenching, η_q=_ quenching yield, *k*_bc_ = rate of back-combination, *k*_ce_ = rate of cage escape, η_ce_ = cage escape yield, *k*_bd_ = rate of back donation, *k*_s_ = rate of SET to the substrate.

Scheme 1 shows an overview of the kinetics associated with the different steps that can take place in photoredox catalysis.^16^ The efficiency of the excitation of the PC to *PC is largely based on the absorption properties of the complex (λ_abs_, ε_max_). In the absence of a suitable quencher (Q), the *PC decays to the GS through radiative (*k*_r_) and nonradiative (*k*_nr_) decay. However, in the presence of Q, outer-sphere quenching can occur through energy transfer (Förster energy transfer and Dexter electron transfer) or, more importantly, through SET. The *PC must be sufficiently long-lived for bimolecular quenching (*k*_q_) to take place, and a high quenching efficiency (η_q_) contributes to the overall efficiency of the reaction. The reduced PC (PC^–^) and oxidized Q (Q^+^) (or vice versa in the case of oxidative quenching of the *PC) are in close contact within the solvent cage. The degree to which they separate (back-combination (*k*_bc_) vs cage escape (*k*_ce_)) is reflected in the cage escape yield (η_ce_). After cage escape, the reduced PC^–^ can go on to either participate in unproductive single electron back-donation to the oxidized quencher (*k*_bd_) or productive SET to a substrate (S) (*k*_s_), which restores the PC to its GS. The electron back-donation can be neglected if the concentration of oxidized quencher is low, that is, if it decomposes more rapidly than electron back-donation takes place. The product Φ = η_q_η_ce_ gives the overall quantum yield for the formation of PC^–^, which corresponds to how many of the absorbed photons generate PC^–^. This highlights the great impact of the cage escape yield and the quenching efficiency for the overall efficiency of the reaction.

### Iron-Based PCs as Alternatives to Traditional TM-PCs

Early examples of TM-driven photoredox catalysis were demonstrated using [Ru(II)(bpy)_3_]^2+^ (**1**) (bpy = 2,2′-bipyridine)^17−20^ and iridium(III) complexes such as [*fac*-Ir(III)(ppy)_3_] (**2**) (ppy = 2-phenylpyridine) and its improved, modified derivative [Ir(III)(dF(CF_3_)ppy)_2_(dtbpy)]^+^ (**3**) (dF(CF_3_)ppy = 2-(2,4-difluorophenyl)-5-(trifluoromethyl)pyridine, dtbpy = 4,4′-di-*tert*-butyl-2,2′-bipyridine).^21−23^ These complexes feature lifetimes of their charge transfer (CT) states in the nanosecond to up to microsecond region. Furthermore, their reactivities and photophysical properties are well-explored. Their successful application is enabled by their strong visible-light absorption and long-lived MLCT states as well as favorable reversible redox chemistry in both their GSs and ESs.^24^ As a result, these noble metal complexes have been successfully employed in a wide range of photocatalytic reactions.^11^ Unfortunately, both these classes of TM complexes harbor the distinct disadvantage of the metals being scarce and expensive, which significantly limits their use for large-scale applications. Additionally, they require irradiation using high-energy light within the blue or violet region of the visible spectrum.^24^
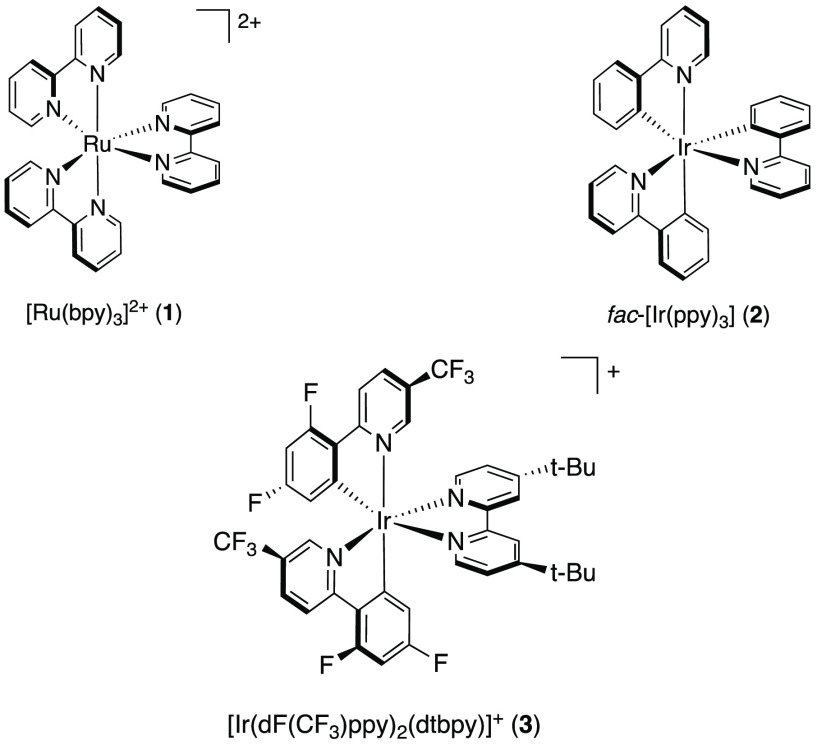


To make photoredox catalysis using TM complexes more environmentally benign and inexpensive, a need for photoactive Earth-abundant metal complexes has arisen, due to their lower cost and comparatively high availability.

When looking at the relative abundance of TMs, especially 3d metals, one element in particular stands out—iron.^25^ The group 8 congeners of said Ru(II)-PCs, based on Fe(II), would present a natural candidate when seeking Earth-abundant alternatives. They should at first glance be a viable option, due to them exhibiting similarly intense visible-light absorption and MLCT transitions.^26^ While there are examples of iron compounds being explored as viable alternatives to precious metal complexes in photoredox catalysis of organic reactions, their number is still small compared to their noble metal competitors. This can be attributed to some of the challenges presented by the photophysical and photochemical properties of iron-based compounds.^27^

**Figure 2:**
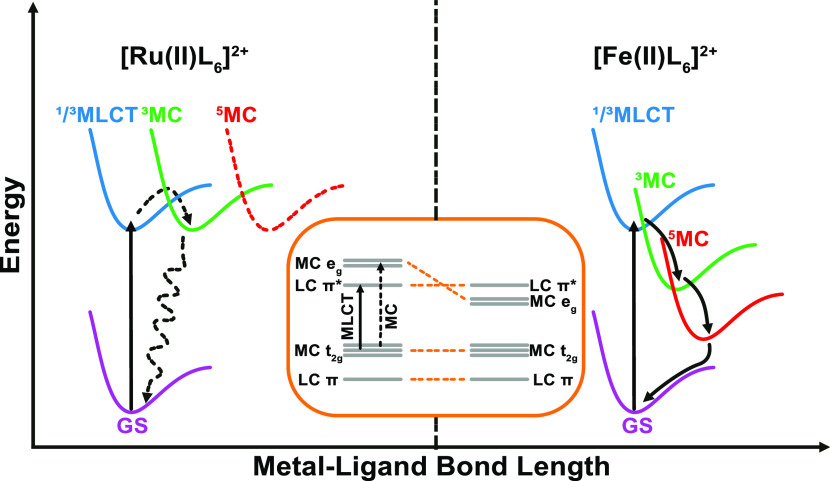
Schematic ES energies of Ru(II)L_6_ and Fe(II)L_6_ complexes. Adapted with permission from ref (27). Copyright 2016 ACS.

Upon direct comparison of the relative energies of the involved ^1/3^MLCT, ^3^MC, and ^5^MC states of Ru(II) and Fe(II) polypyridyl complexes, inherent differences leading to the less favorable properties of the latter are visible (Figure 2). The energies of the e_g_ orbitals and resulting MC states are low-lying, which can be attributed to the comparatively small ligand field (LF) splitting. This in turn opens up for a fast deactivation pathway (100 fs) of the ^3^MLCT state to the low-energy high-spin ^5^MC state, which limits their ability to participate in bimolecular quenching reactions. The lifetime of the MLCT state in these Fe(II) complexes is by a factor of 10^6^ shorter than that of corresponding Ru(II) complexes, which is particularly interesting as these compound classes are isoelectronic.^26^ The short lifetime of the MLCT state and the low energy of the MC state negatively influence the successful application of Fe-PCs in photoredox chemistry. Furthermore, the lack of emission from the MC state limits the photophysical investigations that can be performed on such reaction systems.

Nevertheless, due to the predominant advantages,^25^ many research groups have put efforts into developing various strategies to successfully employ iron photoredox catalysis. This makes it a diverse, up-and-coming field of research with much room for future developments.

In this Perspective, we lay out the challenges faced in iron photoredox catalysis and present selected approaches that have been explored so far—including their benefits and drawbacks—as well as most recent breakthroughs and where we see the future of this rapidly growing research area.

## Utilizing Outer-Sphere Electron Transfer Involving MC States of Iron Photoredox Catalysts

Despite the previously mentioned limitations associated with the MC states of Fe(II) polypyridyl complexes, they have been investigated and utilized as PCs in a variety of photoredox reactions.

### Visible-Light-Mediated Photoredox Catalysis Using [Fe(II)(bpy)_3_]Br_2_ as PC

**Scheme 2:**
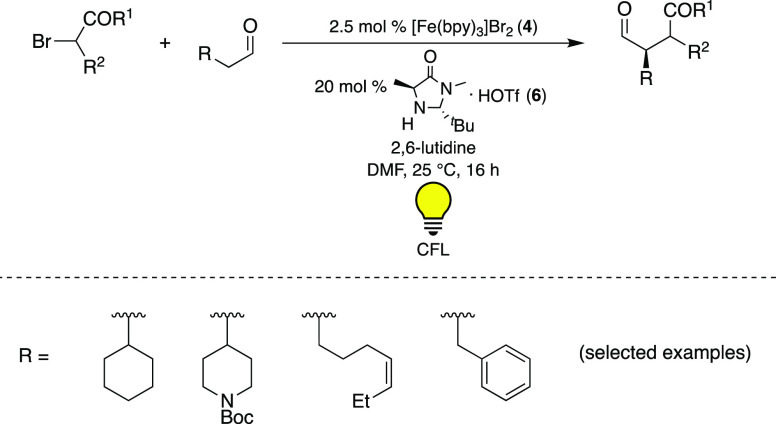
Visible-Light-Mediated Enantioselective Alkylation of Aldehydes Using [Fe(bpy)_3_]Br_2_ (**4**) as PC^28^

In 2015, Cozzi and co-workers demonstrated the seemingly first example of an Fe(II) polypyridyl complex, [Fe(bpy)_3_]Br_2_ (**4**), acting as PC in an organic reaction.^28^ There, the enantioselective alkylation of different aldehydes was studied (Scheme 2)—a reaction that had previously been reported only with traditional noble metal light-harvesters as PCs.^29^
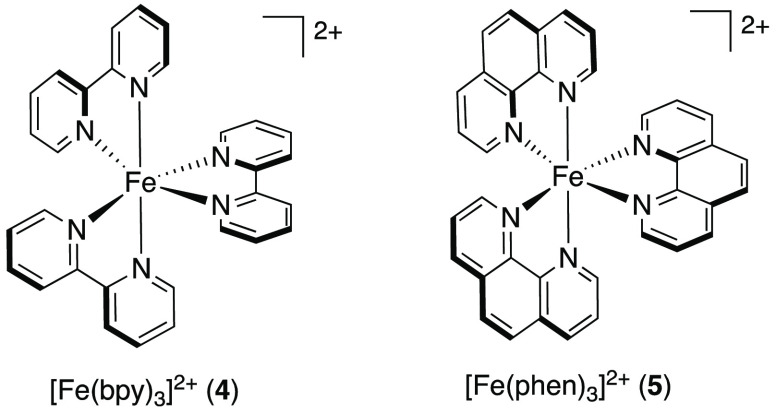


It was found that the reaction proceeded most efficiently when using 2.5 mol % PC and 20 mol % of MacMillan’s imidazolidinone catalyst (**6**)^29^ under visible-light irradiation (23 W CFL (compact fluorescent lamp)). Screening of different iron compounds (Table 1) further revealed that no or close to no conversion of the model substrate, 2-phenylacetaldehyde, was achieved when employing FeBr_2_ or [(PPh_3_)_2_Fe(NO_2_)_2_], respectively, as potential PCs. With [Fe(phen)_3_]Cl_2_ (phen = 1,10-phenanthroline) (**5**) a yield of 89% was obtained, which could be further increased to 92% when [Fe(phen)_3_](PF_6_)_2_ was used as PC. Still, the best yield (99%) was afforded with [Fe(bpy)_3_]Br_2_ (**4**).

**Table 1:** Screening of Different Iron Compounds as PC for the Visible-Light-Mediated Enantioselective Alkylation of 2-Phenylacetaldehyde with Dimethyl Bromomalonate (2.5 mol % PC, 20 mol % (**6**))

PC	Yield (%)
[Fe(bpy)_3_]Br_2_	99
FeBr_2_	0
[(PPh_3_)_2_Fe(NO_2_)_2_]	5
[Fe(phen)_3_]Cl_2_	89
[Fe(phen)_3_](PF_6_)_2_	92

The enantioselectivity of the alkylation is effected by the organocatalyst (**6**) and does not depend on the choice of PC. This was further corroborated by the screening of a range of bromo-substituted carbonyl compounds as substrates, giving similar yields and enantioselectivities for Fe-PC **4**, compared to its noble metal counterparts. The presence of alkene groups in the aldehyde substrate did not lead to any side reactions. All this illustrates the synthetic utility of this reaction.

**Scheme 3:**
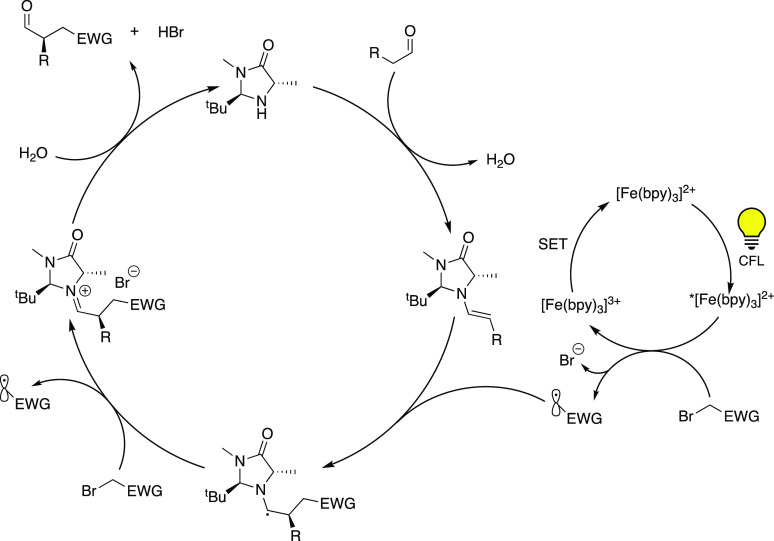
Visible-Light-Mediated Enantioselective Alkylation of Aldehydes Using [Fe(bpy)_3_]Br_2_ (**4**) as PC Adapted with permission from ref (28). Copyright 2015 ACS.

The comparable efficiencies and selectivities of the Fe-PCs in this study establish them as viable alternatives to more common Ru and Ir complexes for use in organic transformations. The Fe-PC fulfills essentially the same role as the noble metal complexes previously used in this particular reaction as indicated in the suggested mechanism in Scheme 3, namely, absorption of a photon resulting in an ES being generated from which a subsequent electron transfer to the alkyl halide occurs. The thereby obtained radical anion then rapidly forms an alkyl radical that would go on to react with the organocatalyst, initiating product formation.

However, as was also acknowledged by Cozzi and co-workers, there are several studies^30−34^ where the photophysical properties of Fe(II) polypyridyl complexes have been investigated indicating that the mechanism of the SET from the excited PC to the substrate is fundamentally different in nature than the mechanism observed for the analogous Ru complexes.^26^

It is well-known that [Fe(bpy)_3_]Br_2_ (**4**), while exhibiting an MLCT band in the visible region, suffers from ultrafast deactivation to its nonluminescent high-spin MC state (650 ps).^34^ While electron injection from the MLCT state of such Fe complexes into TiO_2_ has been demonstrated,^35,36^ it is presumed that there is a limited opportunity for the bimolecular electron transfer to an acceptor molecule to occur in solution, as supported by literature reports.^37,38^

Therefore, several experiments to elucidate the reaction mechanism were performed, including changing the irradiation source from a CFL to a setup that provides lower-energy light (>420 nm), to exclude possible excitation of the reactants by UV irradiation. Electron paramagnetic resonance (EPR) spectroscopy was utilized to detect the presence of radical species during the reaction. This was further supported by control experiments in the presence of TEMPO ((2,2,6,6-tetramethylpiperidin-1-yl)oxyl), which resulted in the reaction not taking place. A radical chain propagation mechanism was proposed to be operative because the reaction continued to proceed even when irradiation was stopped.

In light of these results, and along with the fact that other iron-based compounds such as FeBr_2_ (Table 1) showed no notable reactivity, it is likely that an outer-sphere electron transfer from the excited Fe-PC to the substrate takes place.

### Visible-Light-Mediated Photoredox Catalysis Using [Fe(II)(phen)_3_](NTf_2_)_2_ as PC

**Scheme 4:**
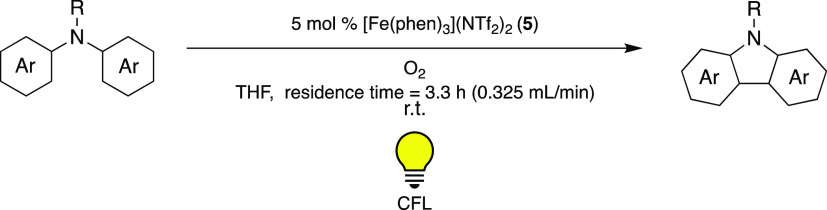
Visible-Light-Mediated Synthesis of Carbazoles Using [Fe(phen)_3_](NTf_2_)_2_ (**5**) as PC and O_2_, under Flow Conditions^39^

Following the aforementioned first successful application of an Fe(II) polypyridyl-based PC in a synthetically useful organic reaction, Collins and co-workers demonstrated the photochemical synthesis of carbazoles using [Fe(phen)_3_](NTf_2_)_2_ (Tf = trifluoromethylsulfonyl) (**5**) and O_2_ in 2016 (Scheme 4).^39^

This reaction was performed under continuous flow conditions and could be used to produce the desired products on gram scale. The yields reported therein were even higher than in previous studies using a Cu-PC, [Cu(I)(Xantphos)(neo)]BF_4_ (Xantphos = 4,5-bis(diphenylphosphino)-9,9-dimethylxanthene, neo = neocuproine), and I_2_.^40^ Different Fe(II) polypyridyl complexes as well as the archetypal [Ru(II)(bpy)_3_]^2+^ (**1**) and [Ir(III)(ppy)_3_] (**2**), the organic dye Eosin Y, and the aforementioned Cu-PC were compared using 5 mol % PC under visible-light irradiation (23 W CFL). The highest yields were obtained using [Fe(II)(terpy)_2_]^2+^ (terpy = 2,2′:6′,2′′-terpyridine) (**7**) (54%) and the even more efficient [Fe(II)phen_3_]^2+^ (**5**) (74%). The lowest yield was observed for the Ir-PC, and yields below 50% were also noted for the Ru-PC, Eosin Y, and the copper complex.
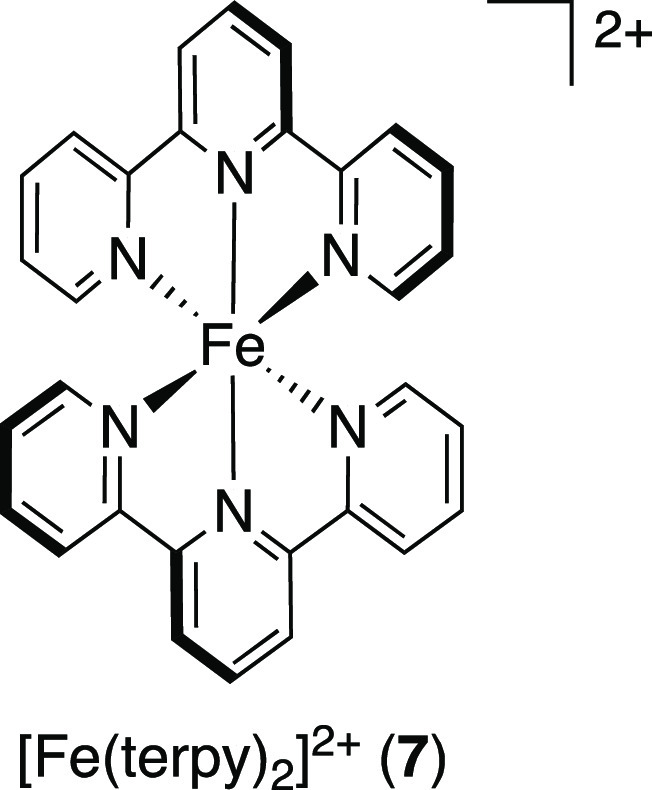


The utility and efficiency of the reaction were demonstrated by converting a range of diaryl- and triarylamines into the corresponding carbazoles. Overall, good to excellent yields were obtained for substrates harboring different functionalities and structural features. Compared to the previously published system using the Cu-PC and I_2_, the yields and catalytic efficiencies in this work were thus significantly improved, showcasing that usage of Fe-PCs can result in superior results to the ones obtained by more common photocatalysts.

Similarly to the work performed by Cozzi and co-workers,^28^ the authors again address the fact that the lifetimes of the MLCT states of Fe(II)-polypyridyl complexes do not allow the photoredox catalysis to operate via the same SET mechanism observed in, e.g., their Ru(II) analogues.

### Mechanistic Investigations of the SET Involving Fe(II) Polypyridyl Complexes

The previously mentioned examples of the successful application of especially Fe(II) polypyridyl complexes constitute one possibility of how to use iron-based PCs for photoredox catalysis via SET.

As was already discussed, the photophysical properties of Fe(II) polypyridyl complexes differ significantly from the ones observed for their second- and third-row TM counterparts.^26^ Due to a lack of insight into the mechanistic details of the aforementioned reactions,^28,39^ investigations toward elucidating their mode of reactivity were needed. These studies can provide an in-depth understanding of the underlying mechanisms, which is crucial for the improvement and optimization of PCs.

**Scheme 5:**
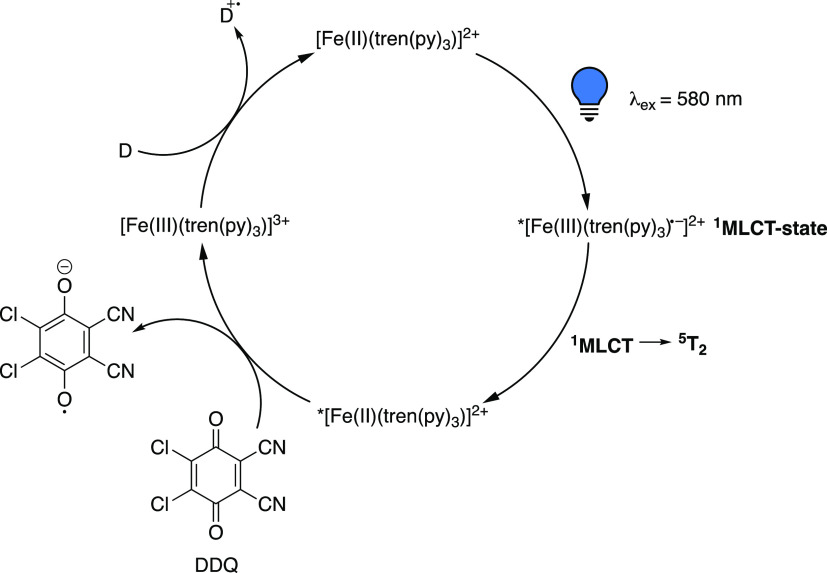
Proposed Mechanism for the Oxidative Quenching of [Fe(II)(tren(py)_3_)]^2+^ (**8**) by DDQ Via SET from the ^5^T_2_ State Following MLCT-MC Conversion D = electron donor. Adapted with permission from ref (41). Copyright 2020 ACS.

Following this line of reasoning, Woodhouse and McCusker^41^ were able to demonstrate that, as a result of the rapid radiationless decay of the ^3^MLCT state of the model compound [Fe(II)(tren(py)_3_)]^2+^ (tren(py)_3_ = tris(2-pyridyl-methylimino-ethyl)amine) (**8**) (200 fs), electron transfer to a quenching reagent, here a range of benzoquinones, takes place from the significantly longer-lived ^5^T_2_ (MC) state (55 ns) (Scheme 5). This is in line with the fact that the formation of the ^5^MC state is orders of magnitude faster than the time scale of diffusion-controlled processes, further supporting the notion that any bimolecular quenching processes would occur from the MC and not an MLCT state.
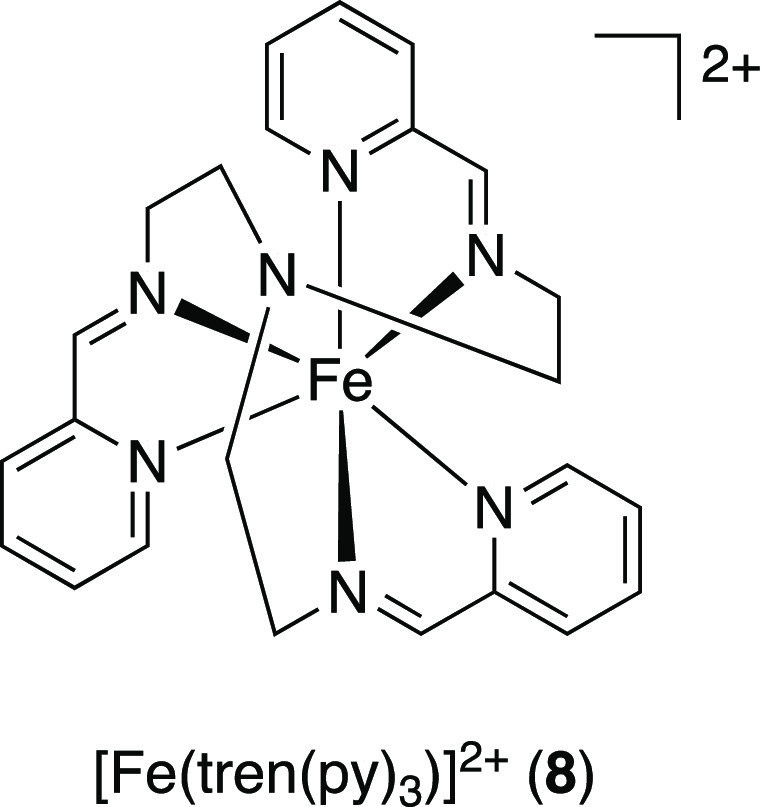


Utilizing Stern–Volmer quenching studies and time-resolved absorption spectroscopy, the authors corroborated such an SET event from the MC state, signifying that the mechanism operating in these types of Fe-PCs is fundamentally different from the CT state-driven photoredox chemistry observed in Ru- and Ir-PCs. The latter states usually exhibit lifetimes of up to microseconds and can store up to 2 eV of energy.^42^ The quenching studies were performed using electron acceptors based on benzoquinones, and the kinetics of the quenching reaction, including the bimolecular quenching rate, were established. While Stern–Volmer plots generally are used to demonstrate a reaction between the ES of a photofunctional molecule and the respective quenching reagent, they do not reveal through which mechanism the electron transfer takes place. Having ruled out an energy transfer or exchange mechanism based on the photophysical properties of the PC under investigation, the electron transfer mechanism remained to be probed. Such an investigation is conventionally achieved through spectroscopic identification of the oxidized/reduced electron donor/acceptor. However, due to an overlap of the absorption profile of the ^5^MC state of the Fe-PC, [Fe(tren(py)_3_)]^2+^ (**8**), and the semiquinone obtained after electron transfer of the quenching reagents, the accurate assignment of species was not unambiguous using electronic absorption spectroscopy. An increase in the concentration of DDQ (DDQ = 4,5-dichloro-3,6-dioxo-1,4-cyclohexadiene-1,2-dicarbonitrile) gradually shortened the lifetime of the ^5^MC state, further providing support for the electron transfer originating from this state as opposed to the CT state. The authors rationalize the presence of an electron transfer pathway based on the observed quenching dynamics and the reduction potentials of the benzoquinones used as quenchers. This was further supported by the fact that the estimated effective ES oxidation potential of the MC state ^5^T_2_ of **8** (ca. −0.35 ± 0.05 V vs Fc^+/0^ in acetone, ca. −0.25 ± 0.05 V vs Fc^+/0^ in acetonitrile) was dependent on the choice of solvent, which is highly indicative of an electron transfer mechanism as opposed to an energy transfer.

Overall, this study highlighted the substantial mechanistic differences in photocatalysis between Fe(II) polypyridyl complexes and their group 8 congeners. The resulting insights regarding the difference in mechanism from their noble metal analogues are of great importance for the optimization and development of this research area, where Fe(II) polypyridyl complexes are used. For the already existing complexes, the redox chemistry of their MC states is considerably less favorable than what could be expected of a sufficiently long-lived CT state, meaning that the scope of possible applications of Fe(II) polypyridyl complexes as PCs is quite limited.

However, this investigation on the bimolecular quenching of the ES in Fe(II) polypyridyl complexes could impact the approach to structural designs for novel iron-based PCs employing MC states. Improvements could, for example, be achieved by destabilizing the e_g_* orbitals using strong field ligands, resulting in MC states of higher energy.

### Visible-Light-Mediated Photoredox Catalysis Using [Fe(III)(acac)_3_] as PC

Another iron complex that has been shown to be useful for the visible-light-induced catalysis of an organic reaction is the photostable^43^ [Fe(III)(acac)_3_] (acac = acetylacetonate) (**9**).
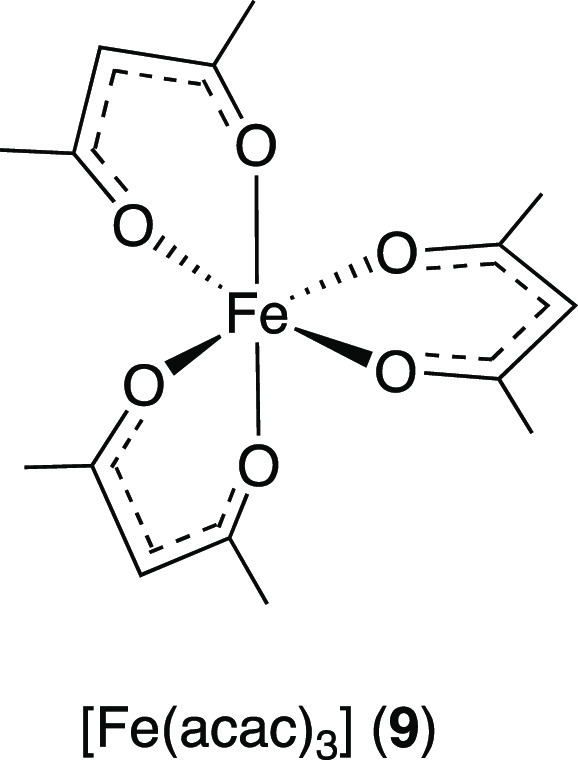


**Scheme 6:**
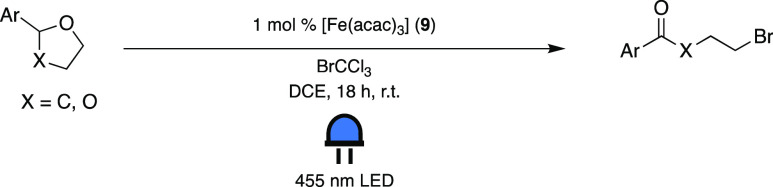
Visible-Light-Mediated Oxidative Fragmentation of Ethers and Acetals Using [Fe(acac)_3_] (**9**) as PC^44^

Recently, Wallentin and co-workers^44^ employed this complex to drive the photocatalytic oxidative ring opening of unstrained cyclic ethers and acetals with unprecedented efficiencies, producing γ- and δ-bromoketones in moderate to excellent yields. They found that their reactions proceeded most efficiently using 1 mol % of [Fe(III)(acac)_3_] (**9**), 3 equiv of BrCCl_3_, 1,2-dichloroethane (DCE) as solvent, and irradiation at 455 nm (Scheme 6). Their methodology was applicable to a range of tetrahydrofuran derivatives with different electron-rich and electron-poor aromatic functionalities, cyclic ethers, and acetals with aromatic and heteroaromatic moieties. Additionally, a tetrahydrofuran derivative with an alkyl substituent as well as acyclic ethers were converted to the corresponding products. Most of these reactions proceeded to give the desired products in moderate to excellent yields over a reaction time of 18 h at room temperature.

In attempts to elucidate the mechanism of these reactions, the authors conducted several control experiments using different PCs and various additives, with, e.g., [Ru(bpy)_3_](PF_6_)_2_ (**1**) giving only 31% yield. A screening of different metal additives showed that Co(ClO_4_)_2_ was also capable of fully converting the model substrate, 2-(4-chlorophenyl)tetrahydrofuran. FeBr_3_ as a PC gave 55% yield, whereas consistent yields of about 90% were only obtained using **9**. Furthermore, the absence of light, both at room temperature and 80 °C, resulted in little to no product being formed in the presence of **9**.

**Scheme 7:**
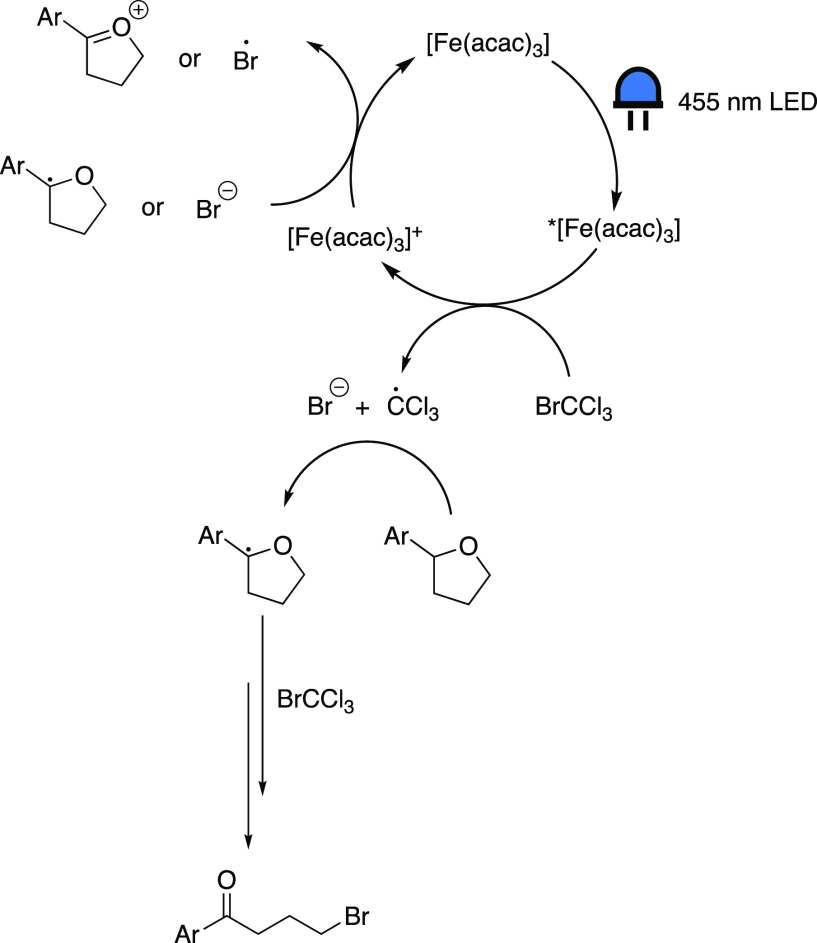
Proposed Mechanism for the Visible-Light-Mediated Oxidative Fragmentation of Ethers and Acetals Using [Fe(acac)_3_] (**9**) as PC Adapted with permission from ref (44). Copyright 2022 ACS.

The following mechanism (Scheme 7) for the reaction was proposed based on the fact that the ES of **9** is likely to activate BrCCl_3_. This conclusion was drawn after demonstrating the formation of the dimerization product, hexachloroethane, as a result of mesolytic cleavage of the halide. Such reactivity suggested that the excited PC is quenched by BrCCl_3_ via SET, after having experimentally supported that the alternative energy transfer mechanism resulting in homolytic cleavage is not operative. Consequently, the authors of this study rationalized that the Fe(III)-PC is excited to its ^4^LMCT state (*E*_1/2 red_ = −1.06 V vs Fc^+/0^),^45,46^ which would be reducing enough to transfer an electron to BrCCl_3_ (*E*_1/2 red_ = −0.56 V vs Fc^+/0^).^11,46^ Thus, they reasoned that structurally simple Fe(III) complexes can drive photochemical reactions from their LMCT states. However, one of the lower-lying MC states of **9** could also be sufficiently reducing (*E*_1/2 red_ = −0.60 V vs Fc^+/0^)^44,46^ to effect this cleavage and is more likely to be involved, as the LMCT states of such *tris*-acetylacetonate d^5^ TM complexes are known to suffer fast deactivation.^43,45^ Nevertheless, it is still noteworthy that, even though the GS is restored after only 50–60 ps after excitation, this complex is capable of driving a reaction—although the exact mechanism of this electron transfer remains unclear.

Taking into account the high possibility of MC state-driven chemistry similar to Fe(II) polypyridyl complexes being in operation here, one very obvious possible drawback of using [Fe(acac)_3_] (**9**) as PC is again the diminished reducing power of the MC state, as compared to the CT state, limiting the possible range of application. Consequently, alternative approaches for iron-based photocatalysis should be explored, as will also be discussed in the following sections of this Perspective.

## In Situ-Generated Photoreactive Iron Complexes

### Visible-Light-Induced Homolysis (VLIH)

**Figure 3:**
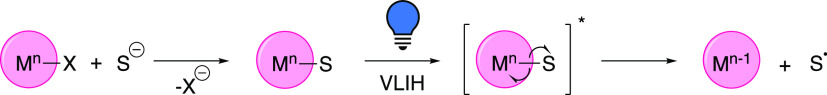
General mode of action for an inner-sphere visible-light-induced homolysis (VLIH) process; S = substrate.

To avoid the problems associated with limited ES-lifetimes necessary for intermolecular electron transfer, one can employ an inner-sphere SET process between the iron center and an in situ-coordinated substrate.^47−49^ This process is more commonly referred to as visible-light-induced homolysis (VLIH)^49,50^ and is initiated by the formation of a metal–substrate complex (Figure 3). Upon photoexcitation, an inner-sphere electron transfer takes place through a CT state, inducing the cleavage of the metal–substrate bond and the generation of a radical substrate species, which is then able to react further. Besides the advantage of avoiding intermolecular, diffusion-controlled CT processes that strongly depend on the sufficiently long ES lifetimes of the PC, the VLIH process is claimed to be advantageous due to its high chemo- and site selectivity.^49,50^

Ordinary MLCT and LMCT excitations in TM complexes generally do not result in homolytic cleavage of the metal–ligand bond. However, in the case of VLIH, the LMCT state induces homolysis of the metal-substrate bond by either depopulation of the σ-bond between the metal and the ligand or population of the antibonding σ*-orbital.^49,50^

**Scheme 8:**
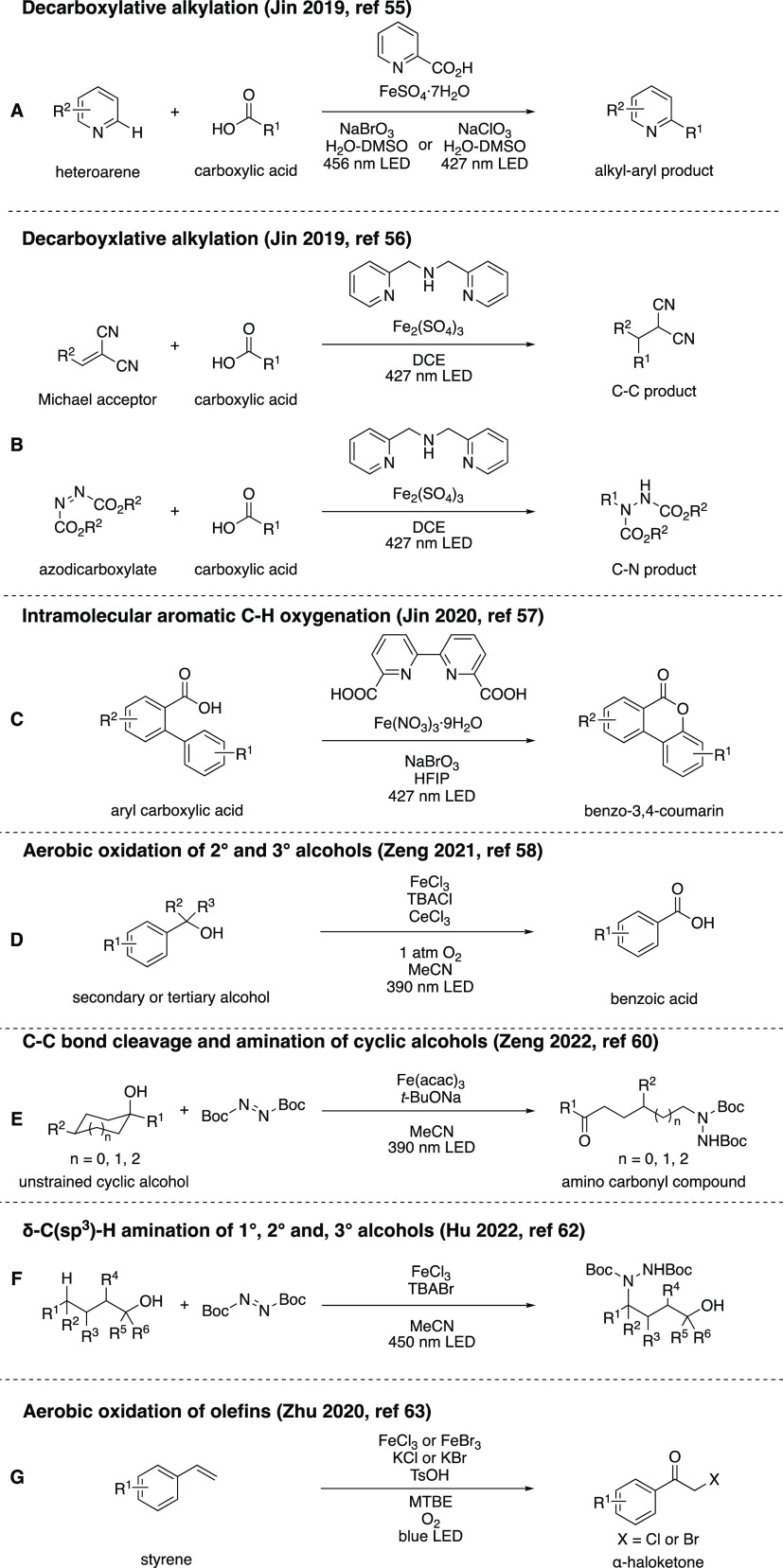
Overview of Inner-Sphere CT Photoredox Reactions Employing Iron

VLIH can be used to catalyze organic reactions by the generation of substrate radicals using a wide variety of Earth-abundant metal–substrate complexes, such as complexes based on nickel, vanadium, cerium, cobalt, copper, and more importantly those based on iron.^49,50^ This section serves to illustrate and discuss a selection of iron-based photoredox catalytic systems (Scheme 8) that operate, or are presumed to operate, via this mechanism.

### Decarboxylative Alkylations

The first example of VLIH involving iron was reported by Parker in 1953, introducing the well-known actinometer based on the generation of ferrous oxalate and carbon dioxide upon irradiation of potassium ferrioxalate.^51,52^ In 1986, Sugimori reported the decarboxylative alkylation of pyridine rings with alkanoic acids, in the presence of a stoichiometric amount of Fe_2_(SO_4_)_3_.^53,54^ Building on these previously established reactions, in 2019, Jin and co-workers catalyzed decarboxylative alkylations of heteroarenes in the presence of FeSO_4_·7H_2_O with 10 mol % of 2-picolinic acid as ligand and sodium bromate or chlorate as oxidant, under irradiation with visible light (Scheme 8A).^55^ Their subsequent report covers a similar protocol, using Fe_2_(SO_4_)_3_ with di-(2-picolyl)amine as a ligand, which was employed to decarboxylatively alkylate Michael acceptors (C–C bond formation) and azodicarboxylates (C–N bond formation) (Scheme 8B).^56^ The benefit of this procedure is that no oxidizing additive is needed, as the radical intermediates are oxidizing enough to oxidize Fe(II) back to Fe(III).

### Intramolecular Aromatic C–H Oxygenation

**Scheme 9:**
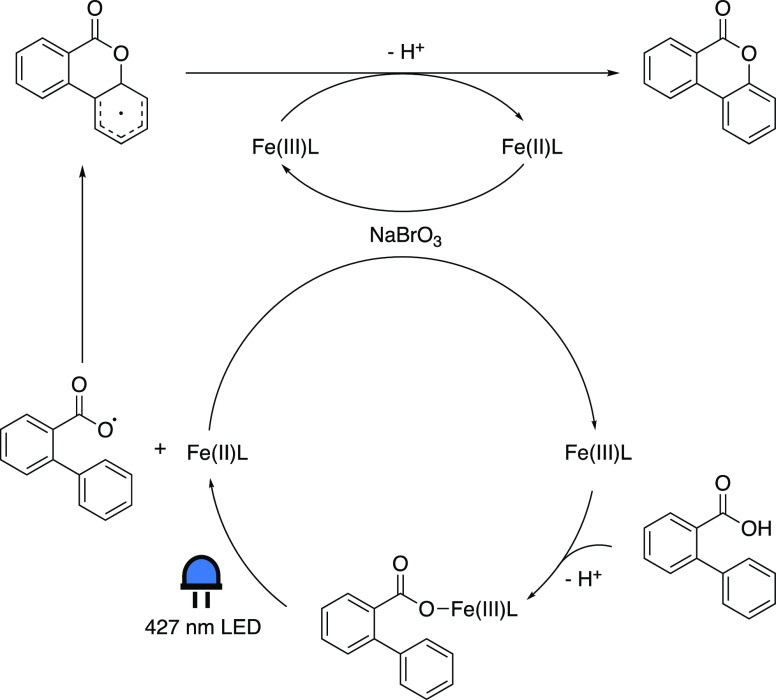
Proposed Mechanistic Cycle of the Intramolecular Aromatic C–H Oxygenation Reaction Adapted with permission from ref (57). Copyright 2020 ACS.

The Jin group also explored an intramolecular aromatic C–H oxygenation reaction of 2-biphenylcarboxylic acids (Scheme 8C), using Fe(NO_3_)_3_·9H_2_O together with 2,2′-bipyridine-6,6′-dicarboxylic acid as ligand and two equivalents of sodium bromate as stoichiometric oxidant under blue light irradiation, to synthesize a wide variety of coumarin derivatives.^57^ UV–Vis absorption spectroscopy indicated an increase in the absorbance in the visible region upon addition of the acid substrate and the ligand, as typically found for in situ-formed photoreactive iron complexes. In the proposed mechanism (Scheme 9) a VLIH event takes place after the coordination of the substrate to the iron center. The subsequent ring closure and SET involving the formed Fe(III) carboxylate species give rise to the Fe(II) species, which upon oxidation by sodium bromate regenerates the original Fe(III) state. Radical trapping experiments further corroborated this mechanism.

### Aerobic Oxidation of 2° and 3° Alcohols to Acids Via α-C–C Bond Cleavage

**Scheme 10:**
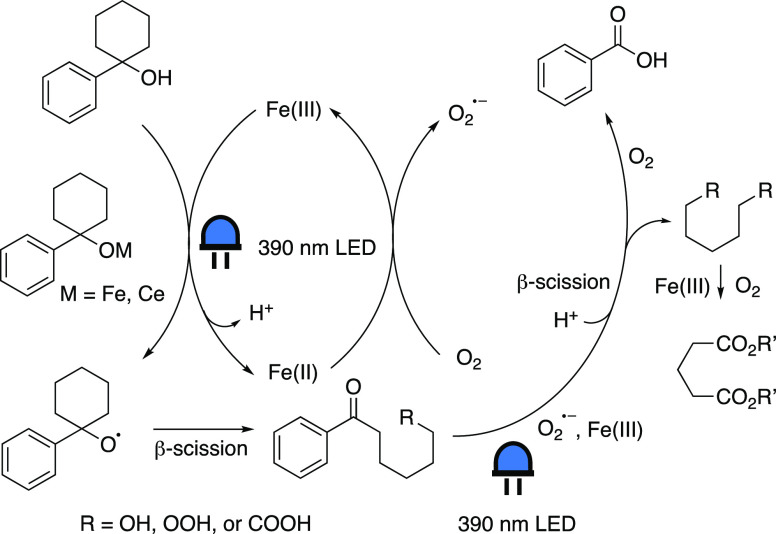
Proposed Mechanism for the Photocatalytic Aerobic Oxidation of Alcohols to Acids Adapted with permission from ref (58). Copyright 2021 ACS.

Zeng’s group employed iron photoredox catalysis for the formation of carboxylic acids from a wide variety of 2° and 3° alcohols (Scheme 8D).^58^ The mode of action (Scheme 10) is proposed to be initiated by coordination of the alcohol oxygen to FeCl_3_, generating a photoactive Fe-alkoxide species, which was supported by UV–vis absorption spectroscopy. After a blue light-induced LMCT event, an Fe(II) species and an O-radical intermediate are allegedly obtained. According to the authors, the iron species is oxidized back to Fe(III) by molecular oxygen, whereas the radical intermediate is proposed to undergo several reaction steps, including a light-induced α-C–C bond cleavage, leading to the desired carboxylic acid product. The addition of CeCl_3_ as a cocatalyst to the system is believed to enhance the efficiency of the photoinduced LMCT step. Furthermore, Fourier transform infrared (FT-IR) spectroscopy was employed to track the formation of the ketone intermediate and subsequent formation of the carboxylic acid functionality. Chemical trapping experiments were performed to confirm the presence of radical intermediates.

### C–C Bond Cleavage and Amination of Unstrained Cyclic Alcohols

**Scheme 11:**
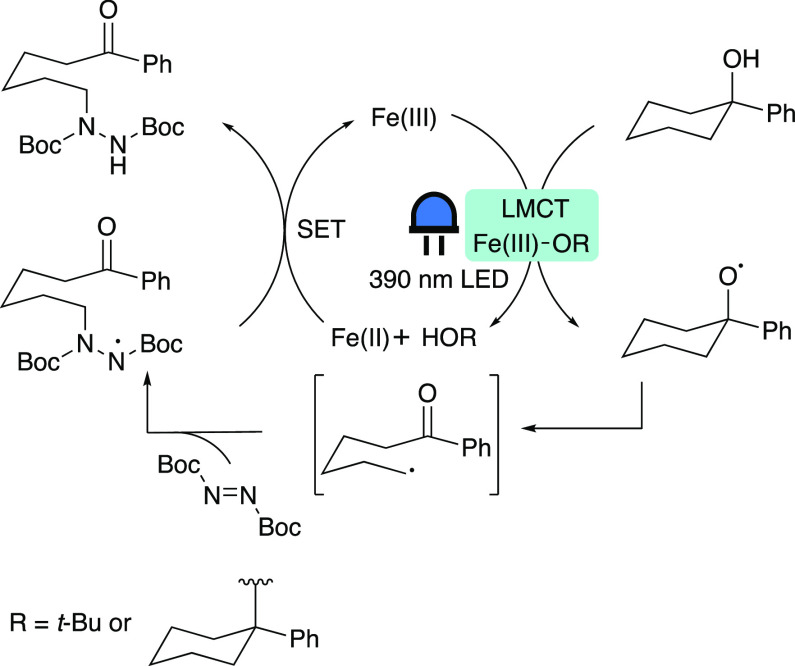
Proposed Mechanism for the Photocatalytic Amination of Unstrained Cyclic Alcohols Adapted with permission from ref (60). Copyright 2022 ACS.

Zeng and co-workers also investigated the halogenation and amination of cyclic and linear alcohols using iron photoredox catalysis.^59−61^ Here, we discuss their report on the photocatalytic amination of unstrained cyclic alcohols (Scheme 8E).^60^ The proposed mechanism (Scheme 11) is initiated by the in situ formation of Fe(O*t*-Bu)_3_ from [Fe(acac)_3_] and *t*-BuONa. This Fe(III) species generates a *tert*-butoxide radical upon the photoinduced (390 nm) LMCT excitation that performs hydrogen atom transfer (HAT) from the cyclic alcohol, leading to the desired alkoxy radical intermediate. Alternatively, the substrate could replace a *tert*-butoxide moiety at the metal center, also leading to the reactive alkoxy radical intermediate. This radical then undergoes β-scission, generating the desired ketone moiety and an alkyl radical, which in turn is trapped by di-*tert*-butyl azodicarboxylate (DBAD). SET from the Fe(II) species and protonation gives the desired aminated product and regenerates the Fe(III) species. The UV–vis absorption spectrum of [Fe(acac)_3_] in acetonitrile exhibited a broadening and red-shift of the absorption maximum upon addition of DBAD, indicating that this substrate might act as a ligand as well. Altogether, the mechanism is not completely elucidated, and the exact nature of the photoreactive species remains unknown.

### δ-C(sp^3^)-H Amination of 1°, 2°, and 3°Alcohols

In a follow-up investigation by Hu’s group, the replacement of [Fe(acac)_3_] by FeCl_3_, as well as addition of TBABr (tetrabutylammonium bromide) instead of *t*-BuONa, allowed for the expansion of the substrate scope to include sterically hindered primary, secondary, and tertiary alcohols (Scheme 8F).^62^ The mechanism for this reaction largely resembles that of the previously mentioned investigation by Zeng and co-workers on the photocatalytic amination of unstrained cyclic alcohols. The key difference lies in the generation of the reactive alkoxy radical intermediate, which is shown to take place both via VLIH, involving the LMCT state of the Fe(III)-OR (R = alkyl) species, as well as the VLIH of FeCl_3_ itself, generating a chlorine radical, which participates in hydrogen abstraction of the alcohol, in this case.

### Aerobic Oxidation of Olefins

**Scheme 12:**
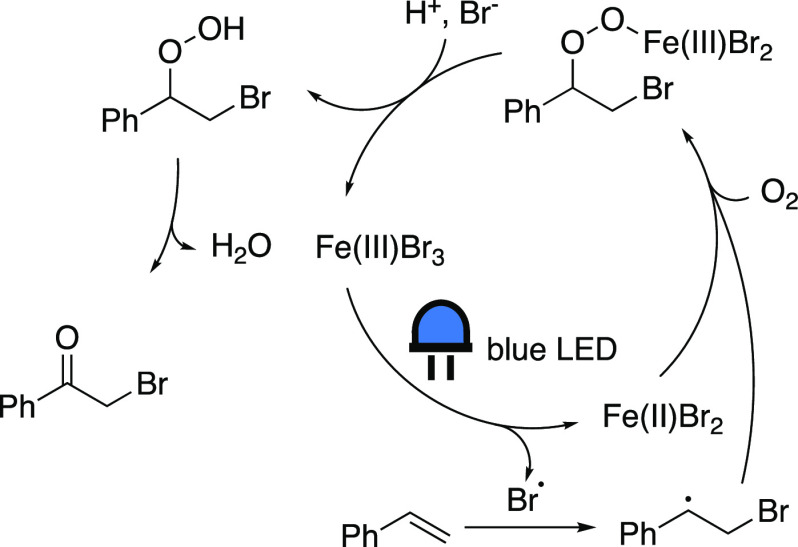
Proposed Mechanism for the Aerobic Oxidation of Olefins Adapted with permission from ref (63). Copyright 2020 Wiley.

Another application of VLIH based on iron was discovered by Zhu’s group, in which α-haloketones are generated by the aerobic oxidation of olefins employing FeBr_3_ (or FeCl_3_) as a catalyst, in the presence of excess potassium halide (Scheme 8G).^63^ The UV–vis spectra of these complexes exhibit an absorption maximum at 430 nm, allowing for photoredox catalysis employing blue light irradiation. In the suggested mechanism (Scheme 12) a bromine radical is generated upon irradiation of Fe(III)Br_3_, which is subsequently trapped by the olefin. This so-formed organic radical intermediate is captured by oxygen, after which the resulting organic peroxide coordinates to Fe(II)Br_2_, oxidizing the iron center to Fe(III). Finally, it is proposed that, in the presence of a bromide source and an acid, the catalyst is regenerated, and a free organic peroxide intermediate is formed, which gives an α-bromoketone as product upon dehydration. The presence of a radical pathway was supported by trapping experiments.

### Formation of Chlorine Radicals to Induce Reactions

**Scheme 13:**
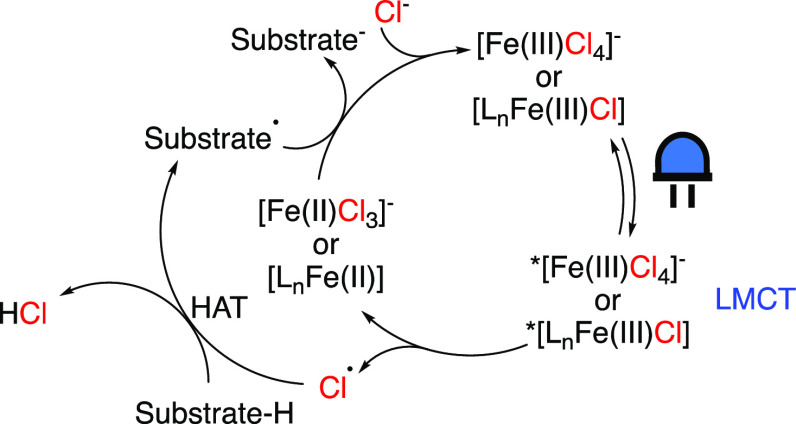
General Catalytic Cycle for Photoredox Catalysis Using Free Chlorine Radicals Generated from a Photoreactive Iron Species

**Scheme 14:**
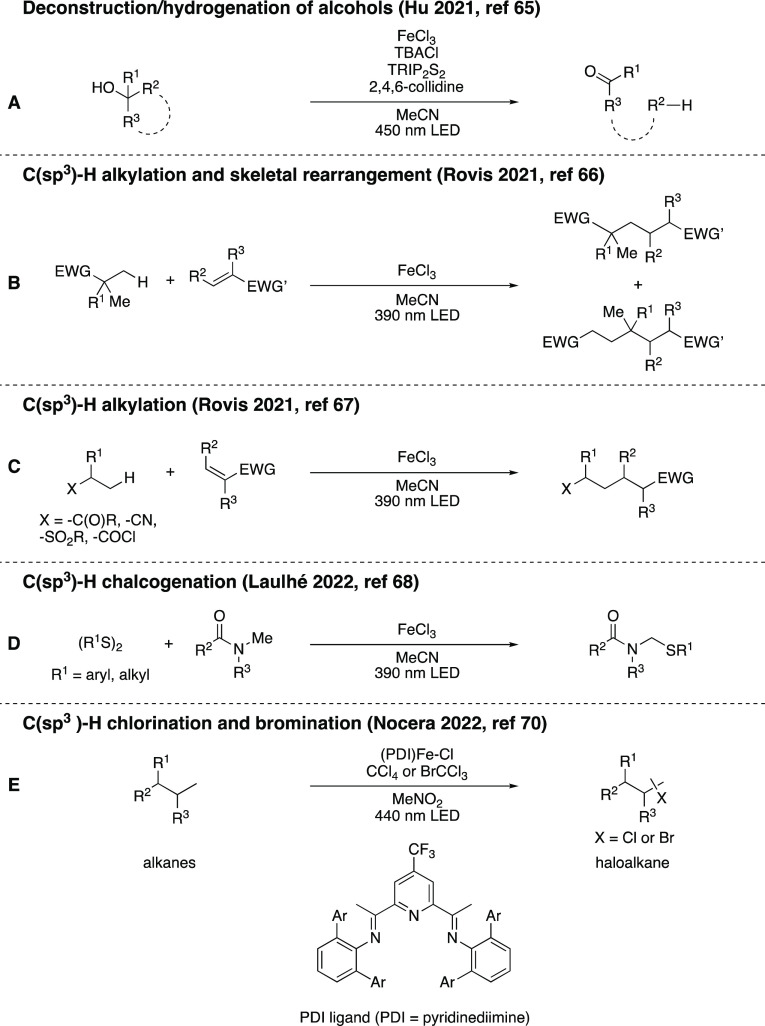
Overview of Inner-Sphere CT Photoredox Reactions Utilizing In Situ-Generated Chlorine Radicals

Another interesting mode of action in photoredox catalysis is the in situ generation of chlorine radicals by a photoinduced LMCT event (Scheme 13). These so-formed chlorine radicals are reactive enough (*E*_ox_ (Cl^–^/Cl^·^) = +1.65 V vs Fc^+/0^)^46,64^ to activate substrates by HAT. The activated substrate can oxidize the iron species, regenerate the catalyst, and form a substrate anion that reacts further. Although this reaction mode is of use in the catalysis of a wide variety of organic transformations,^64−70^ the exact photoreactive species are in many cases not unambiguously assigned by thorough mechanistic investigations. The traditional reaction systems utilizing visible light (Scheme 14A–D)^65−68^ suffer from impaired reactivity in solvents other than acetonitrile and show a general necessity for irradiation with higher-energy light (390 nm). However, the adaption of the reaction system through the addition of additives, such as TRIP_2_S_2_ (1,2-bis(2,4,6-triisopropylphenyl) disulfane) and 2,4,6-collidine (Scheme 14A)^65^ or the introduction of pyridine-diimine (PDI)-based ligands (Scheme 14E)^70^ increases the absorption wavelength to 440–450 nm, resulting in more benign reaction conditions, thereby potentially diminishing undesirable side-reactions. Furthermore, it is noteworthy that the reactivity of the free chlorine radical generally results in poor regioselectivity. Nocera and co-workers recently solved this problem by the introduction of PDI-based ligands, inducing regioselectivity through confinement of the chlorine radicals within the secondary coordination sphere via the formation of a Cl^·^|arene complex with the arene moieties present in the PDI-based ligands.^70^

### Conclusion on VLIH Employing Iron

VLIH based on in situ-formed iron complexes has already now found applications in the field of organic chemistry, and more applications could be foreseen in the future. However, there are significant drawbacks associated with this strategy: Although the substrate scopes for the specific reactions are generally broad, the range of reactions that this type of chemistry can drive appears limited, making this mode of photoredox catalysis not widely employable. It is necessary for the substrate to coordinate to the iron center, and the in situ-formed complex must allow for visible-light absorption, electron transfer, and subsequent homolytic cleavage. This makes it challenging to strategize adaptations and consequently “tune” the photoreactive species to fit a wider variety of reaction types. Furthermore, although chlorine radicals have been shown to drive a range of organic transformations by employing the widely available FeCl_3_, regioselectivity remains a common issue. Since using such a reactive species and employing irradiation at shorter wavelengths could lead to unwanted side reactions, further improvements, such as those explored by Nocera and co-workers,^70^ are necessary.

### Other In Situ-Formed Fe Complexes (non-VLIH)

**Scheme 15:**
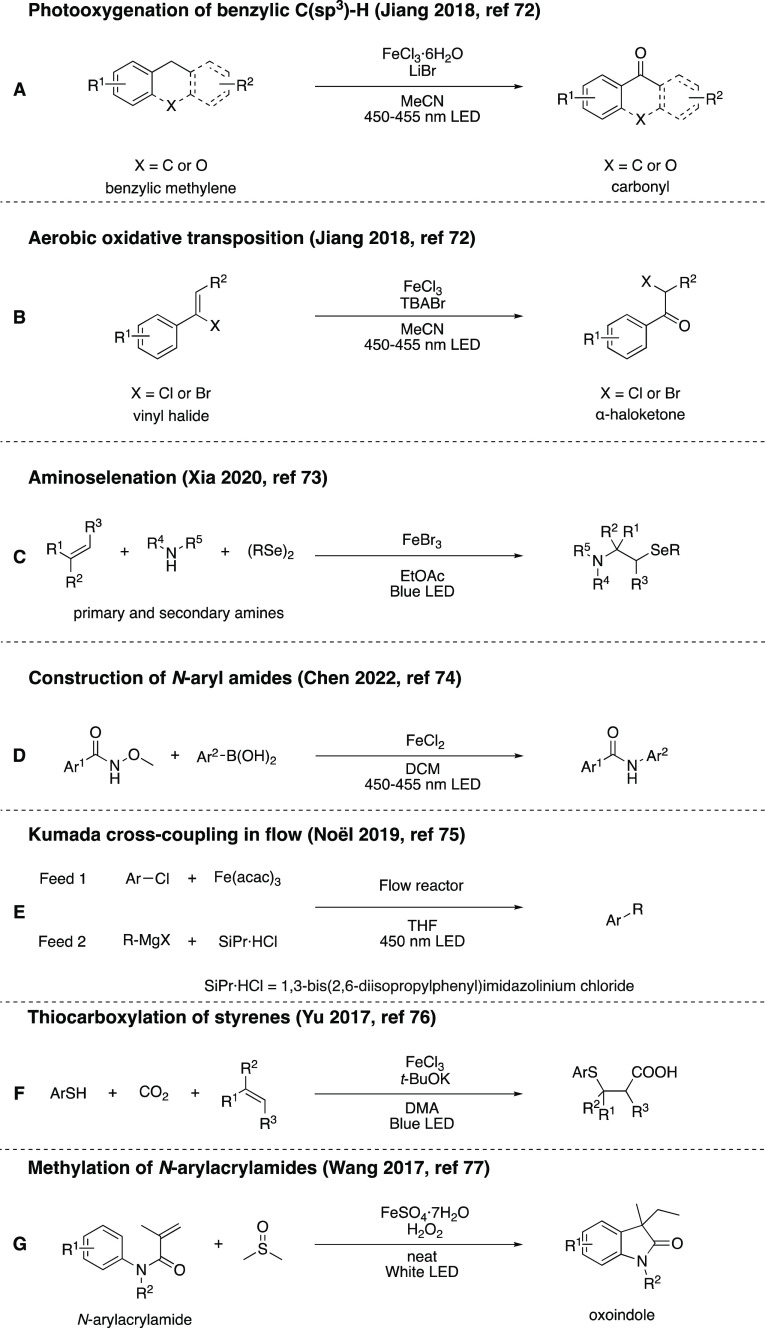
Overview of Reactions Utilizing In Situ-Generated Iron Catalysts Operating Via Mechanisms Other than VLIH

Other examples of utilizing in situ-formed iron complexes not employing VLIH have also been developed, albeit few in number (Scheme 15).^71^ It is noteworthy that less-thorough mechanistic investigations have been performed on reactions driven by non-VLIH catalysis as compared to their VLIH counterparts.

Jiang and co-workers reported photooxidation of benzylic C(sp^3^)-H bonds (Scheme 15A) as well as the aerobic oxidative transposition of vinyl halides (Scheme 15B) by in situ-generated tetrahalogenoferrate(III) complexes, using blue light.^72^ Xia’s group investigated the blue-light-driven aminoselenation of alkenes employing FeBr_3_ as a precatalyst in air (Scheme 15C).^73^ Chen and co-workers reported a photoinduced iron-catalyzed electrophilic amidation for the construction of *N*-aryl amides (Scheme 15D).^74^ Noël, Alcázar, and co-workers demonstrated C(sp^2^)-C(sp^3^) Kumada-Corriu cross-coupling reactions in flow, promoted by visible light in the presence of [Fe(acac)_3_], an NHC ligand (SIPr-HCl = 1,3-bis(2,6-diisopropylphenyl)imidazolinium chloride), and Grignard reagents (Scheme 15E).^75^ Yu and co-workers proposed a thiocarboxylation of styrenes, in the presence of FeCl_3_, under visible-light irradiation (Scheme 15F).^76^ Lastly, Wang and co-workers reported the methylation of *N*-arylacrylamides with dimethyl sulfoxide (DMSO) as the methylating agent, to synthesize 3-ethyl-3-methyl oxindoles in a Fenton-type reaction, using FeSO_4_·7H_2_O and H_2_O_2_ under irradiation with white light-emitting diode (LED) light (Scheme 15G).^77^

This section exemplifies again the synthetic utility of in situ-formed photoreactive iron species. Although there are benefits associated with using such iron species, such as the inexpensiveness and simplicity of the added iron compounds, these strategies are currently restricted to a limited number of organic transformations. Furthermore, as the publications covering these types of reactions feature few mechanistic investigations of the underlying photocatalytic processes, they provide limited contributions to an improved understanding of the field of iron photoredox catalysis as a whole.

## Application of the Charge-Transfer State of Iron Complexes in Photocatalysis

**Figure 4:**
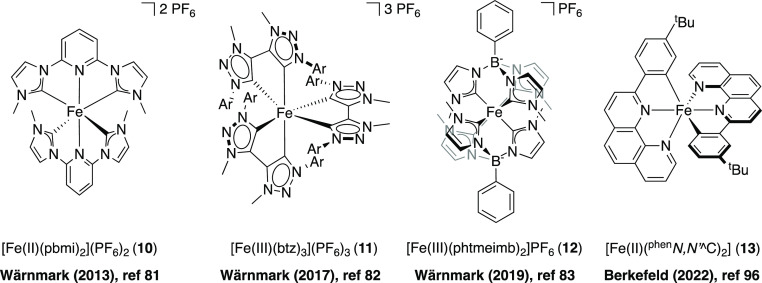
(left to right) The first Fe-NHC complex with an extended CT ES lifetime (9 ps) and the three iron complexes with demonstrated photoactive CT ESs to date (0.1–2 ns). Ar = *p*-tolyl.

As mentioned in the preceding section, utilizing inner-sphere CT processes can circumvent the challenges associated with the short ES lifetimes of iron(II) polypyridyl complexes. An alternative approach is to improve the CT lifetimes of the iron complexes themselves, allowing for more efficient outer-sphere electron transfer.^78−80^ Several strategies have been employed to make the MLCT states, and eventually LMCT states, more long-lived, such as improving the octahedral geometry of the complex to increase the overlap between ligand orbitals and the d_*z*^2^_ and d_*x*^2^–*y*^2^_ orbitals of iron, thus increasing the energy of the e_g_ set of orbitals, which disfavors deactivation of the excited state via MC states. Increased rigidity of the ligand backbone to limit geometric reorganization in the excited state is another strategy to slow down nonradiative decay.^78,80^ The arguably most impactful strategy to date has been the incorporation of strongly σ-donating *N*-heterocyclic carbenes (NHCs), initially in cooperation with π-accepting pyridine substituents. The first example utilizing this design strategy was demonstrated by the synthesis of the tetra-NHC complex [Fe(II)(pbmi)_2_](PF_6_)_2_ (**10**) (pbmi = (pyridine-2,6-diyl)bis(1-methyl-imidazol-2-ylidene)) by Wärnmark and co-workers in 2013, which exhibits a ^3^MLCT lifetime of 9 ps (Figure 4).^81^ In a subsequent investigation, [Fe(btz)_3_](PF_6_)_3_ (btz = 3,3′-dimethyl-1,1′-bis(*p*-tolyl)-4,4′-bis(1,2,3-triazol-5-ylidene)) (**11**) was discovered as the first luminescent iron complex harboring an extended photoinduced ES lifetime (100 ps) in solution at room temperature, owing to the presence of three bidentate mesoionic NHC ligands.^82^ This was promptly followed by the synthesis of the bis-tridentate scorpionate Fe(III) complex [Fe(phtmeimb)_2_](PF_6_) (**12**) (phtmeimb = phenyl(tris(3-methylimidazol-2-ylidene))borate), which features an increased ES lifetime of 2.0 ns in acetonitrile.^83^ This complex contains two scorpionate ligands with a total of six NHCs in the form of imidazolines as well as two negatively charged boron atoms, enhancing the electron donation even further. It is worth noting that complex **12** possesses superior photostability compared to [Ru(bpy)_3_]^2+^ (**1**). As a consequence of the strong σ-donation and lack of π-accepting pyridines in the NHC-ligands, the GSs for both Fe complexes **11** and **12** changed from Fe(II) to Fe(III) under ambient conditions, which led to a change in the ES from a ^3^MLCT to a ^2^LMCT state. Furthermore, they exhibit favorable absorption maxima in the green region of the visible spectrum. These interesting features have made these two iron complexes attractive targets for exploration of their functionality as PCs in visible-light photoredox catalysis.^84−88^

In the initial investigation of [Fe(phtmeimb)_2_]^+^ (**12**), its ES was shown to be oxidatively quenched by the methyl viologen dication as well as reductively quenched by diphenylamine. Bimolecular quenching rate constants in acetonitrile were determined from Stern–Volmer plots of emission lifetimes τ_0_/τ, showing a diffusion-controlled quenching rate in the case of diphenylamine (*k*_q_ = 1.4 × 10^10^ M^–1^ s^–1^) and only a slightly lower quenching rate for the methyl viologen dication (*k*_q_ = 2.7 × 10^9^ M^–1^ s^–1^).^83^ These initial quenching studies were then followed up by a more thorough investigation of the reductive quenching by the amine electron donors triethylamine (TEA) and dimethylaniline (DMA).^89^ These studies showed equally ultrafast diffusion-controlled quenching by DMA (*k*_q_ = 2.75 × 10^10^ M^–1^ s^–1^) and almost as fast electron transfer from TEA (*k*_q_ = 8 × 10^9^ M^–1^ s^–1^). Notably, the dynamic quenching rate of *[Fe(phtmeimb)_2_]^+^ (*12) in DMA is more than 2 orders of magnitude larger than that of the archetypal [Ru(bpy)_3_]^2+^ (**1**).^90,91^ Unfortunately, ultrafast spin-allowed geminate charge recombination, with rates of *k*_cr_ ≈ 0.2 ps^–1^, occurs for both DMA and TEA, effectively hampering high cage escape yields that would be needed for efficient photocatalysis. As we will discuss in the next section, high-yielding photocatalysis has, however, been possible with **12** as a result of clever modification of reaction conditions.^84,85^

### The First Example of Photoredox Catalysis Driven by an Fe-NHC Complex

**Scheme 16:**
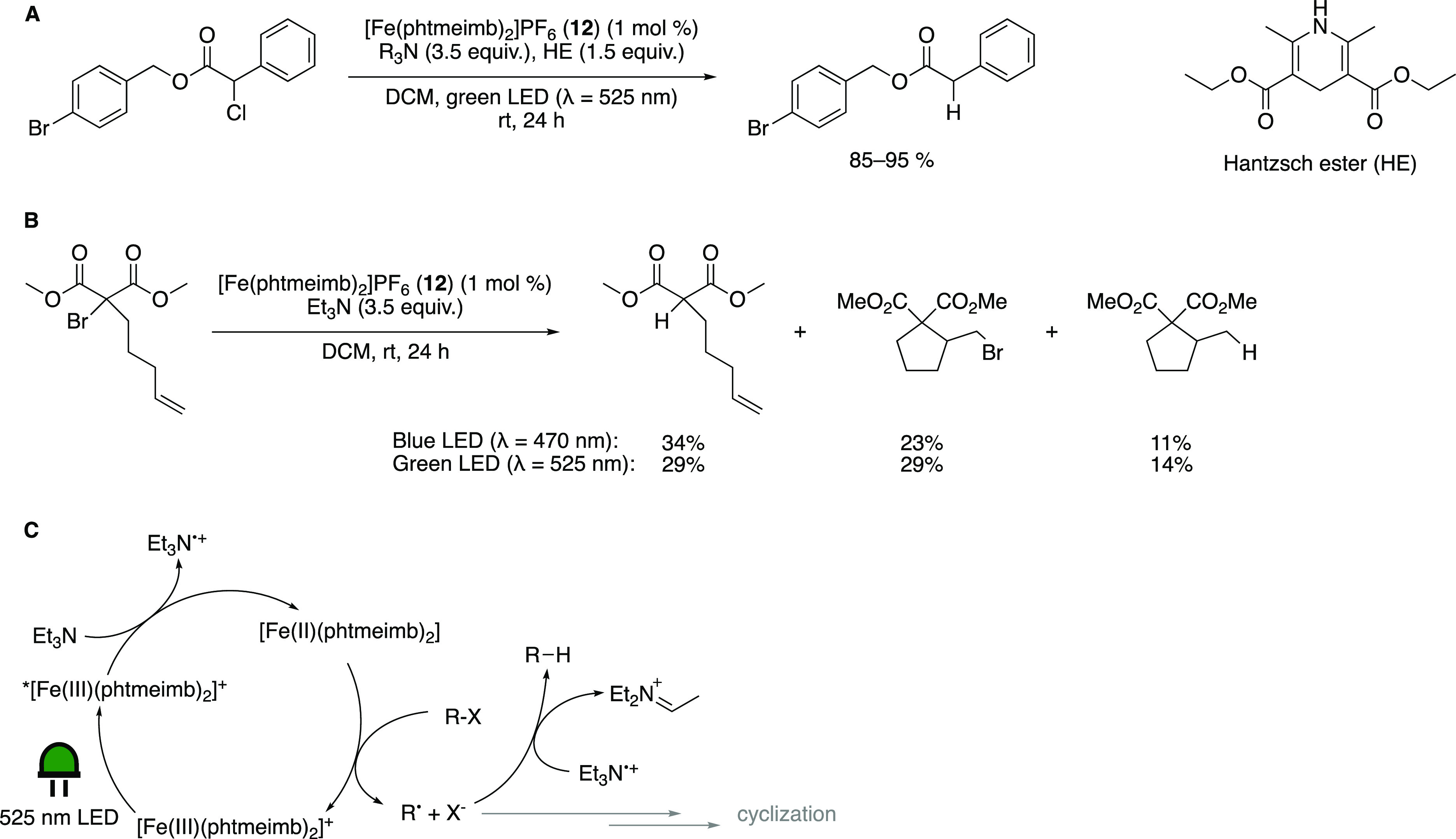
(A) Dehalogenation Reaction Studied by Troian-Gautier and Co-Workers; R_3_N = Amine Used as Sacrificial Reductant. (B) Follow-up Dehalogenation/Cyclization Reaction by Troian-Gautier and Co-Workers. (C) General Mechanism of the HAT Reaction Adapted with permission from refs (84) (Copyright 2021 ACS) and (85) (Copyright 2021 RSC).

The first study utilizing an Fe-NHC complex as a PC was published in 2021 by Troian-Gautier and co-workers. There, they explore the use of [Fe(phtmeimb)_2_]PF_6_ (**12**) in a dehalogenation reaction with Hantzsch ester (HE) as an HAT reagent and a variety of amines as sacrificial electron donors (Scheme 16A), employing green light irradiation.^84^ The lifetimes of *12 in different solvents were determined to range between 1.7 and 2.4 ns. The rate constants for the quenching of *12 by a wide range of amines in the different solvents were also measured. The Stern–Volmer plots were linear, consistent with a dynamic quenching mechanism, meaning that it is the ES that is interacting with the quencher. As previously shown for other quenchers, the quenching rate constants were close to the diffusion limit and ranged *k*_q_ = (0.86–2.65) × 10^10^ M^–1^ s^–1^ for the aromatic amines, and *k*_q_ = (0.03–1.89) × 10^10^ M^–1^ s^–1^ for the aliphatic amines. Electron transfer from the quencher to *12 was supported by transient absorption spectroscopy. The cage escape yield was found to be largely solvent-dependent, with η_ce_ = 0.36–0.63 in dichloromethane (DCM), and η_ce_ = 0.01–0.07 in acetonitrile and dimethylformamide (DMF). The authors suggest that the high η_ce_ in DCM most likely is due to a combination of two different effects. First, the heavy-atom effect can induce state-mixing, conveying a partial spin-forbidden character to the geminate charge recombination, decelerating it and thereby improving the η_ce_.^16^ Second, the observed increase in η_ce_ can also be a result of solvent dielectric effects in combination with the electrostatic repulsion between the reduced PC and the oxidized electron donor.

The aforementioned efficient quenching of *12 by amines facilitated the model dehalogenation reaction shown in Scheme 16A. Interestingly, at higher concentrations of aliphatic amines such as TEA, no additional HE was needed, pointing to the fact that these amines can act as HAT reagents themselves (Scheme 16C).

Although higher quenching efficiencies and cage escape yields were obtained for tertiary aromatic amines than for aliphatic amines bearing α-hydrogens, lower yields of the dehalogenated products were obtained. A potential explanation is that the oxidized tertiary aromatic amine and the reduced photocatalyst can undergo fast charge recombination, even after cage escape. However, amines with α-hydrogens rapidly decompose irreversibly, effectively hindering charge recombination.^92^ This indicates not only that the quenching yield and cage escape yield matter but also that charge recombination can still play a role after cage escape.

In a follow-up study by the same group, a mechanistic investigation of another dehalogenation and a cyclization reaction (Scheme 16B) was published.^85^ The same trend of higher cage escape yields in DCM than in other solvents was also observed here. Interestingly, the use of [Ru(bpy)_3_](PF_6_)_2_ (**1**) and [Ir(ppy)_2_(bpy)]PF_6_ as PCs gave exclusively the cyclized products under otherwise identical conditions, whereas **12** gave a mixture of all three possible products, indicating the involvement of different mechanisms for the iron and noble metal photocatalysis.

As Troian-Gautier and co-workers demonstrated, it is possible to increase η_ce_ of [Fe(phtmeimb)_2_]PF_6_ (**12**) by a careful selection of solvent and additives. However, as recently shown in a study of **12** in the context of a hydrogen evolution reaction (HER), low quenching efficiency can still be counteracted by employing high concentrations of the quencher, resulting in high TONs.^86^

In a very recent publication by the group of Troian-Gautier,^93^ complex **12** and its dibromo-substituted analogue ([Fe(Br-phtmeimb)_2_]^+^),^94^ along with other TM complexes, were investigated as PCs for the borylation of aryldiazonium salts under irradiation (525 nm). Although comparatively poor yields and conversions were obtained for both the Fe-PCs, this report showcases yet another example of the application of iron compounds for photoredox catalysis.^93^

### Photocatalysis Driven by the Relatively Short-Lived CT States of [Fe(btz)_3_](PF_6_)_3_

**Scheme 17:**
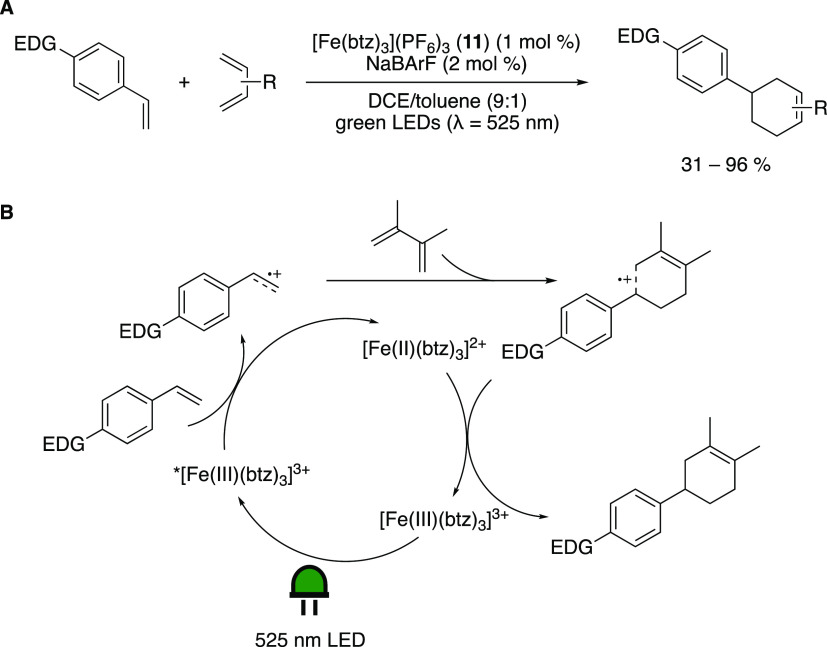
(A) Radical Cationic [4 + 2] Cycloaddition Driven by Green Light Irradiation of [Fe(btz)_3_](PF_6_)_3_ (**11**). (B) Mechanism proposed by Kang and Co-Workers Adapted with permission from ref (87). Copyright 2022 ACS.

Due to the relatively short ^2^LMCT lifetime (τ = 100 ps) of the mesoionic carbene complex [Fe(III)(btz)_3_](PF_6_)_3_ (**11**, Figure 4),^82^ the bimolecular quenching had not been studied. However, as shown by the group of Kang in 2022, complex **11** can act as a PC. This Fe-PC exhibits an absorption maximum at 558 nm, and its ES (*11) is a strong oxidant (*E*°(*Fe(III)/Fe(II))) = 1.60 V vs Fc^+/0^),^82^ which is used to effectively drive a radical cationic [4 + 2] cycloaddition reaction between electron-rich styrenes and various dienes under green light irradiation (Scheme 17).^87^ The reduction potential of *11 matches well with the oxidation potential of terminal styrenes (*E*_ox_ = 0.9–1.3 V vs Fc^+/0^). Addition of a small amount of NaBArF (sodium tetrakis[3,5-bis(trifluoromethyl)phenyl]borate, 2 mol %) as a noncoordinating anion additive and the cation-stabilizing cosolvent toluene proved beneficial to the reaction. The methodology was applied to a wide range of *para*-substituted styrenes in combination with different dienes, including some late-stage derivatization of natural products, in fair to very good yields. The reaction is proposed to proceed via SET from the styrene to *11. The oxidized styrene then reacts in a cationic [4 + 2] cycloaddition with the diene. The newly formed radical cation proceeds to regenerate Fe(III)-PC **11** from [Fe(II)(btz)_3_](PF_6_)_2_ (**14**)^95^ via SET. A competing pathway, where singlet oxygen is generated through energy transfer, and then reduced to O_2_^·^^–^, which drives the reaction, is also proposed. The quantum yield of Φ = 0.49 suggests that any radical chain pathway is inefficient.

**Scheme 18:**
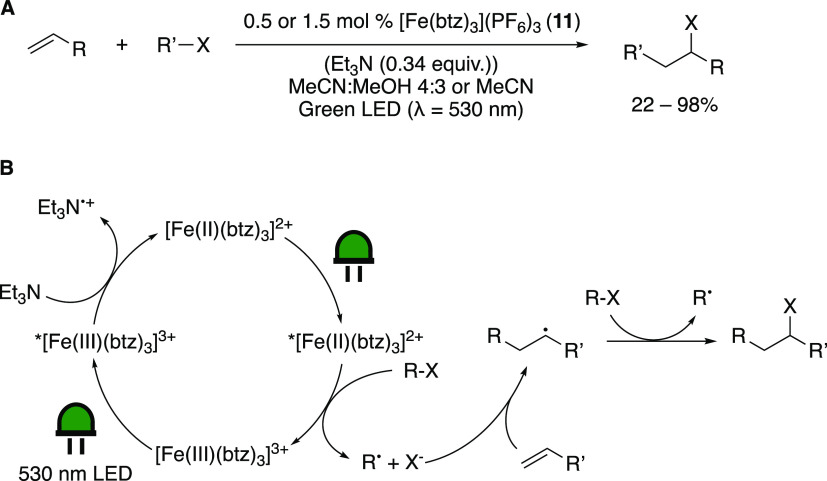
(A) The ATRA Reaction Driven by Green Light Irradiation of [Fe(btz)_3_](PF_6_)_3_ (**11**). Reductive Quenching: 0.5 mol % **11**, TEA (0.34 equiv) in Acetonitrile/Methanol 4:3. Oxidative Quenching: 1.5 mol % **11** in Acetonitrile. (B) The Reaction Mechanism of the Reductive Green-Light-Driven ATRA Reaction Adapted with permission from ref (88). Copyright 2022 RSC.

Concurrently, Wärnmark and co-workers studied the application of [Fe(btz)_3_](PF_6_)_3_ (**11**) in an Atom Transfer Radical Addition (ATRA) reaction (Scheme 18A). Based on the redox potentials of the complex (Table 2), two different possible pathways were envisioned. One is based on oxidative quenching of the excited Fe(III) complex by an alkyl halide, and the other is based on reductive quenching by a sacrificial reductant (TEA). Both reactions were shown to be feasible, and a broad substrate scope was demonstrated, showing a wide tolerance of different functional groups, including an addition to an alkyne and an intramolecular cyclization. The reductive quenching cycle is suggested to proceed via a two-photon catalytic cycle involving both photoactive oxidation states of the complex, in a consecutive photoinduced electron transfer (Scheme 18B)—a reaction mode rarely observed for photoredox catalysis employing TMs but reminiscent of a Z-scheme also found in photosynthesis. The obvious advantage is that higher reduction potentials are reached that might not be accessible in a single photon process.

**Table 2:** GS and ES Properties of Fe(III) Complexes Used in CT State-Driven Photoredox Catalysis, in Comparison with a Traditional Ir(III)-PC^c^

Complex	[Fe(III)(btz)_3_](PF_6_)_3_ (**11**)	[Fe(III)(phtmeimb)_2_]PF_6_ (**12**)	[Ir(III)(dF(CF_3_)ppy)_2_(dtbpy)]PF_6_ (**3**)^a^
Type of ES	LMCT	LMCT	MLCT
λ_abs_ [nm]	558	502	380
ε_max_ [10^3^ · M^−1^ cm^−1^]	1.2	3.0	6.2
λ_em_ [nm]	600	655	470
τ [ns]	0.10	1.96	2300
Φ [%]	0.03	2.1	68
*E*^1^/_2_(M(IV)/M(III)) [V vs Fc^+/0^]	1.2	0.25	1.38
*E*^1^/_2_(M(III)/M(II)) [V vs Fc^+/0^]	–0.58	–1.16	–1.68^b^
*E*°(M(IV)/*M(III)) [V vs Fc^+/0^]	–1.0	–1.88	–1.20
*E*°(*M(III)/M(II)) [V vs Fc^+/0^]	1.60	1.0	0.90
Ref	(82)	(83)	(23)

a The redox potentials for [Ir(III)(dF(CF_3_)ppy)_2_(dtbpy)]PF_6_ (**3**) were converted from V vs saturated calomel electrode (SCE) to V vs Fc^+/0^ using the value for Fc^+/0^ vs SCE of +0.31 V cited in the original paper.^23^

b Value for *E*_1/2_(L/L^–^).

c All redox potentials are given vs ferrocenium/ferrocene (Fc^+/0^) in acetonitrile.^46^ Values for [Fe(btz)_3_](PF_6_)_3_ (**11**) and [Fe(phtmeimb)_2_]PF_6_ (**12**) were obtained in air-saturated acetonitrile at room temperature.

### Novel Cyclometalated Iron Complex with a Luminescent MLCT State

**Scheme 19:**
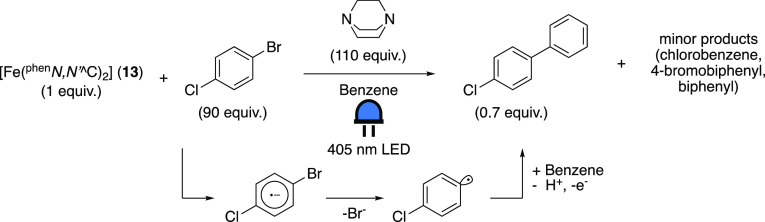
Stoichiometric Photoinduced Aryl–Aryl Coupling Driven by Blue-Light Irradiation of [Fe(II)(^phen^*N,N’*^^^C)_2_] (**13**) Adapted with permission from ref (96). Copyright 2022 ACS.

Recently, an iron complex exhibiting luminescence in the IR region (λ_em_ = 1220 nm) from a ^3^MLCT state was disclosed.^96^ This complex, [Fe(II)(^phen^*N,N’*^^^C)_2_] (^phen^*N,N’*^^^C = 2-(4-(*tert*-butyl)phenyl)-1,10-phenanthroline, **13**, Figure 4), has not yet been proven to be photocatalytically active. However, it is still worth mentioning because it represents an alternative design path for photoactive iron complexes. In addition to the relatively long lifetime of τ = 0.8 ns in benzene, the MLCT state is highly reducing, surpassing the ES of [Fe(phtmeimb)_2_]PF_6_ (**12**) (−2.0 V and −1.9 V vs Fc^+/0^, respectively). The authors furthermore show that it can stoichiometrically photoinduce aryl–aryl coupling between *p*-chloro-bromobenzene and benzene (Scheme 19). Noteworthy is also that the reaction does not seem to proceed via direct excitation from the GS to the MLCT state, since it is driven by blue-light irradiation (λ = 405 nm), and the absorption maximum of the complex is located in the red region (λ = 765 nm). In the photoreaction an unidentified intermediate complex forms, which seems unable to drive the reaction further, causing the characteristic absorption band of [Fe(II)(^phen^*N,N’*^^^C)_2_] (**13**) to disappear.

### Comparing the GS and ES Properties of Photoactive Iron and Noble Metal TM Complexes

Table 2 and Table 3 illustrate the range of different redox potentials accessible by photoactive iron complexes in comparison with conventional noble metal PCs. As can be seen, highly reductive as well as highly oxidative ESs have been achieved.

**Table 3:** GS and ES Properties of Fe(II) Complexes Used in CT State-Driven Photoredox Processes, and a Comparison with a Traditional Ru(II)-PC^f^

Complex	[Fe(II)(btz)_3_](PF_6_)_2_ (**14**)	[Fe(II)(^phen^*N,N’*^^^C)2] (**13**)	[Ru(II)(bpy)_3_]Cl_2_ (**1**)
Type of ES	MLCT	MLCT	MLCT
λ_abs_ [nm]	730	765	452
ε_max_ [10^3^ · M^−1^ cm^−1^]	1.22	–a	14.6
λ_em_ [nm]	–a	1220	625
τ [ns]	0.528	0.8^b^	180 | 930^c^
Φ [%]	–a	–a	1.8 | 9.5^c^
*E*^1^/_2_(M(III)/M(II)) [V vs Fc^+/0^]	–0.58	0.88	0.92
*E*^1^/_2_(L/L^–^) [V vs Fc^+/0^]	–2.38	–2.46	–1.69^d^
*E*°(M(III)/*M(II)) [V vs Fc^+/0^]	–1.6	–2.0	–1.20
*E*°(*L/L^–^) [V vs Fc^+/0^]	–1.4^e^	–1.36	0.34
Ref	(95)	(96)	(42,97,98)

a Not determined in the paper.

b Measured in benzene.

c Measured in air-saturated and oxygen-free acetonitrile solution, respectively.

d Measured in methanol.

e Irreversible reduction.

f All redox potentials are given vs Fc^+/0^ in acetonitrile.^46^

## Conclusion and Outlook

Since the report by Cozzi in 2015, an impressive amount of different photoredox reactions using iron PCs have been developed. Three major strategies have emerged since then, namely, (1) outer-sphere electron transfer from MC states, (2) inner-sphere electron transfer, and (3) outer-sphere electron transfer from CT states. Although outer-sphere electron transfer from MC states has found various practical applications, the substrate scope employing such a mechanism is rather limited due to the modest redox potentials MC states exhibit. An alternative strategy, photoinduced inner-sphere electron transfer, has also proven useful for photocatalysis of organic reactions. However, for such a mechanism to work, the substrate must coordinate to the iron center, which restricts the more widespread employment of this approach.

The last and, in our opinion, most promising strategy is the use of new photoactive iron complexes with long-lived CT states. So far, only iron complexes with NHC ligands have been shown to be photocatalytically active via SET from CT states. Besides the synthetic application of these complexes in photoredox catalysis, thorough mechanistic investigations were executed, leading to valuable new insights for further development. For example, the common notion that mainly the ES lifetime is important to obtain high reaction yields is put into question as the relatively short-lived *[Fe(btz)_3_]^3+^ was able to efficiently drive photoredox catalytic reactions. Further studies on the exact influence of catalyst design and solvents on the quantum yields and cage escape yields are, however, necessary. Furthermore, the ^2^LMCT state of Fe(III) complexes could fundamentally impact the observed photochemical reactivity. The shift of absorption toward green wavelengths, in addition to the observed photostability of Fe-NHCs, allowing for mild and prolonged irradiation, thereby counteracting a potentially lower quantum yield, are advantages that remain underutilized to date.^83,84,86^ The dual excitation of an iron complex in two different oxidation states, as was demonstrated in the ATRA reaction using [Fe(II/III)(btz)_3_]^2+/3+^, is another intriguing approach to facilitate thermodynamically challenging reactions.

There has been a surge of Fe-photoredox catalysis in recent years, and we expect this growth to continue. The design of new complexes, by tuning of their ligands, will probably widen the range of accessible redox potentials, both in the GS and the ES, much in the way it has been done with noble metal PCs. However, due to the still rather difficult synthesis of iron complexes with photofunctional CT states, an improvement in reactivity is necessary to justify making iron photoredox catalysis part of the organic chemists’ toolbox. Regardless, some argue iron photoredox catalysis is already more sustainable and cost-efficient than noble metal catalysis.^25^ The field would greatly benefit from more in-depth photophysical investigations, as these can lead to significant insights into this rapidly progressing research area. Traditional reaction optimization under different conditions, improving the photoredox potentials of Fe-PCs through ligand design, and, more importantly, the advancement of the mechanistic understanding of iron-based photoredox reactions by collaborations between researchers in synthetic chemistry and spectroscopy, might open up an entirely new space of reactions.
